# Receptors that bind to PEDF and their therapeutic roles in retinal diseases

**DOI:** 10.3389/fendo.2023.1116136

**Published:** 2023-04-17

**Authors:** Manhong Xu, Xin Chen, Zihao Yu, Xiaorong Li

**Affiliations:** Tianjin Key Laboratory of Retinal Functions and Diseases, Tianjin Branch of National Clinical Research Center for Ocular Disease, Eye Institute and School of Optometry, Tianjin Medical University Eye Hospital, Tianjin, China

**Keywords:** pigment epithelium-derived factor (PEDF), receptor, adipose triglyceride lipase (ATGL), laminin receptor (LR), lipoprotein receptor-related protein 6 (LRP6), plexin domain-containing (PLXDC), F1-ATP synthase, vascular endothelial growth factor receptor 2 (VEGFR2)

## Abstract

Retinal neovascular, neurodegenerative, and inflammatory diseases represented by diabetic retinopathy are the main types of blinding eye disorders that continually cause the increased burden worldwide. Pigment epithelium-derived factor (PEDF) is an endogenous factor with multiple effects including neurotrophic activity, anti-angiogenesis, anti-tumorigenesis, and anti-inflammatory activity. PEDF activity depends on the interaction with the proteins on the cell surface. At present, seven independent receptors, including adipose triglyceride lipase, laminin receptor, lipoprotein receptor-related protein, plexin domain-containing 1, plexin domain-containing 2, F1-ATP synthase, and vascular endothelial growth factor receptor 2, have been demonstrated and confirmed to be high affinity receptors for PEDF. Understanding the interactions between PEDF and PEDF receptors, their roles in normal cellular metabolism and the response the initiate in disease will be accommodating for elucidating the ways in which inflammation, angiogenesis, and neurodegeneration exacerbate disease pathology. In this review, we firstly introduce PEDF receptors comprehensively, focusing particularly on their expression pattern, ligands, related diseases, and signal transduction pathways, respectively. We also discuss the interactive ways of PEDF and receptors to expand the prospective understanding of PEDF receptors in the diagnosis and treatment of retinal diseases.

## 1 Introduction

Pigment epithelium-derived factor (PEDF), also known as serpin family F member 1 (SERPINF1), is a 50 kDa protein encoded by the SERPINE1 gene (1). The human gene encoding PEDF is located on chromosome 17p13.3 and comprises 9 exons encoding a 418 amino acid protein (NCBI Reference Sequence: NG_028180.1). PEDF belongs to the serine protease inhibitor superfamily (SERPINS), the members of which share a common structural element, a reactive center loop (RCL) (2). Most SERPINS members are serine protease inhibitors, including plasmin inhibitors, antichymotrypsin, and antitrypsin (3). However, PEDF is considered to be a noninhibitory serpin as it is activated after chymotrypsin RCL cleavage and lacks protease activity (4).

In 1991, Joyce Tombran-Tink et al. first discovered that medium from retinal pigment epithelial (RPE) cells induced Y79 retinoblastoma tumor cell differentiation and increased tumor cell neurite outgrowth. Considering these findings, they identified the protein with the neurotrophic function induced by RPE cell-conditioned medium and termed it PEDF (1, 5). In addition to exerting as a neurotrophic factor, PEDF was recognized for its other functions. One decade after its discovery, PEDF was shown to protect motor neurons from chronic glutamate-mediated neurodegeneration (6) and protect photoreceptors from light damage (7, 8). In addition, PEDF is considered to be a potent angiogenesis inhibitor, which contributes to its important role in both antiangiogenesis and antitumorigenesis (9, 10). This discovery initiated a new era of PEDF function and mechanism exploration in angiogenic and degenerative diseases, especially in diabetes and diabetic complications.

An increasing number of studies have shown that the PEDF mechanism of action and related molecular pathways are involved in mediating or inducing signal transduction. PEDF activity depends on the high affinity of PEDF for interacting proteins, namely PEDF receptors on the cell surface (11). Understanding the interactions between PEDF and PEDF receptors, their roles in normal cellular metabolism and the response the initiate in disease may help elucidate the ways in which inflammation, angiogenesis, and neurodegeneration exacerbate disease pathology. In this review, we introduce these PEDF receptors to readers in a comprehensive way and suggest the potential underlying mechanisms for diseases characterized by multipathological changes, including angiogenesis, neurodegeneration, and inflammation. This increase in understanding may lead to new approaches to combat these diseases.

## 2 PEDF distribution and expression

The mRNA encoding PEDF (SERPINF1 mRNA) has been identified in several kinds of normal human tissues and organs, including blood cells, plasma, lymph nodes, brain, spinal cord, heart, arteries, muscle, lung, eye, bone, colon, kidney, liver, spleen, breast, thyroid, prostate, and pancreas (12). SERPINF1 mRNA is also expressed in embryonic ocular tissues. PEDF has been found in developing cones, RPE granules, neuroblasts and the ganglion cell layer (GCL) of developing human retinas (13). PEDF can also be found in the human amniotic membrane (14). In embryonic mouse eyes, PEDF is first detected on embryonic day (E) 14.5 in RPE cells, initially in the inner plexiform layer and subsequently, on E18.5, in the GCL layer, inner nuclear layer, choroid and ciliary body (15). In mouse embryonic livers, PEDF is found on E12.5, and gradually increases and maintains high expression in the liver (16). In addition, PEDF is highly expressed in murine primary long-term hematopoietic stem cells (HSCs) (17).

PEDF levels have been shown to be decreased in angiogenic tissues or organs, such as the retinas from patients with proliferative diabetic retinopathy (PDR) (18–24). PEDF exerts antiangiogenic effects by inducing endothelial cell apoptosis (25, 26), which contributes to its antitumor effects. On the other hand, PEDF directly promotes differentiation, inhibits proliferation, mediates tumor cell apoptosis, and ultimately abrogates tumors (12), such as melanomas (27, 28), pancreatic carcinomas (29), prostate tumors (30) and ovarian tumors (31). An increasing number of studies on PEDF have shown that this protein maintains the growth and development of important organs and supports important organ functions. For example, PEDF played cardioprotective roles by increasing glucose uptake in the ischemic myocardium, an effect mediated through PI3K/AKT signaling and GLUT4 translocation (32) and enhanced cardiac function by reducing hypoxia-induced cell injury (33). Pulmonary PEDF expression was increased in idiopathic pulmonary fibrosis and inversely correlated with pulmonary microvascular density and vascular endothelial growth factor (VEGF) levels (34). Plasma PEDF levels in chronic kidney disease patients were found to be significantly increased compared with those in patients without chronic kidney disease (35). In addition, renal PEDF levels were significantly decreased in a diabetic rat model (36). Exogenously overexpressed PEDF in mice resulted in the inhibition of retinal neovascularization (RNV) in an oxygen-induced retinopathy (OIR) model (37). However, PEDF deficiency caused severe vessel loss in the retinas of the OIR model mice (38).

The PEDF receptors discovered to date include patatin-like phospholipase domain-containing protein 2 (PNPLA2)/adipose triglyceride lipase (ATGL) (39), laminin receptor (25), lipoprotein receptor-related protein 6 (LRP6) (40), plexin domain-containing 1 (PLXDC1), PLXDC2 (41), F1-ATP synthase (42), and vascular endothelial growth factor receptor 2 (VEGFR2) (43). In this review, we expound on the distribution and function of each PEDF receptor, as well as the molecular signaling pathways in which they are involved (see **Table 1**).

**Table 1:** PEDF receptor summary.

PEDF Receptors	Other Names	Chr	Expression in Tissues	Expression in Cells	Ligands	Related Diseases	Reference
**ATGL**	PNPL2 iPLA2ζ Desnutrin	11	adipose tissue, bone marrow, colon, and small intestine	human Y-79 cell, mouse photoreceptor cells (661W), rat photoreceptor cell line (R28), and bovine retinal RPE cells	PEDF, LC3, ABHD5, UBXD8, G0S2, HIG2, TNF-α, insulin, PPAR-γ, PKA, AMPK, and Fsp27	Insulin resistance Fatty liver Inflammation	(44–47)
**LRP6**	ADCAD2 STHAG7	12	heart, brain, placenta, spleen, testes, and ovaries	human hippocampus, rental tubular cells, hepatocytes, intestinal epithelial cells, osteoblasts, osteoclasts, and developing embryo cells	PEDF, Wnt ligands, and Fzd	Brain defects Posterior truncation Abnormal limb patterning Alzheimer’s disease Atherosclerosis Diabetes Hyperlipidemia Hypertension Coronary artery disease	(48–55)
**PLXDC1**	TEM3 TEM7	7	gall bladder, endometrium, ovary, heart, esophagus	endothelial cells and rodent ganglion cells	PEDF, LRP1, and Nidogen	Glioblastoma Ovarian cancer Gastric cancer Renal cell carcinoma Colorectal cancer Lung cancer Breast cancer Osteosarcoma	(56–64)
**PLXDC2**	TEM7R	10	gall bladder, lung, skin, endometrium, kidney	human tumor vascular endothelial cells, neural progenitor cells, pluripotent stem cells, and hepatocytes	PEDF, Wnt3a, Wnt5a, Wnt8a, and ADGRD1	Coronary artery disease Ovarian cancer Diabetic mellitus Diabetic retinopathy Testicular germ cell tumors Sotos syndrome Ischemic stroke Laryngeal squamous cell Carcinoma Breast carcinoma Breast cancer Colorectal tumor	(56, 65–74)
**LR**	LAMR RPSA	3	ovary, lymph node, bone marrow, appendix, esophagus	tumor cells and blood cells	PEDF	Breast cancer Lung cancer Ovarian cancer Prostate cancer Gastric cancer Thyroid cancer Leukemia Lymphoma	(75, 76)
**F1-ATP Synthase**	ATP5F1	18 5	heart, kidney, duodenum, colon, adrenal	endothelial cells, hepatocytes, adipocytes, and keratinocytes	PEDF and F0-ATP Synthase	Angiogenesis diseases Lipoprotein metabolism diseases Innate immunity diseases Hypertension Food intake dysregulation	(77)
**VEGFR2**	Flk-1 KDR CD309	4	lacenta, thyroid, fat, lung, endometrium	endothelial cells, hematopoietic cells, macrophages, and smooth muscle cells	PEDF and VEGFA	Tumor angiogenesis	(78, 79)

Chr, Chromosome; PNPL2, Patatin-Like Phospholipase Domain Containing 2; iPLA2ζ, Independent Phospholipase A2-ζ; ADCAD2, Coronary Artery Disease, Autosomal Dominant 2; STHAG7, Selective Tooth Agenesis 7; TEM3, Tumor Endothelial Marker 3; TEM7, Tumor Endothelial Marker 7; TEM7R, Tumor Endothelial Marker 7-Related Protein; LAMR, Laminin Receptor; RPSA, Ribosomal Protein SA; Flk-1, Fetal Liver Kinase 1; KDR, Kinase Insert Domain Receptor; CD309, Cluster of Differentiation 309; ATP5F1; ATP Synthase F1; LC3, Light Chain 3; ABHD5, α/β Hydrolase Domain-Containing 5; UBXD8, UBX Domain-Containing Protein 8; G0S2, G0/G1 Switch 2; HIG2, Hypoxia-induced Gene 2; TNF-α, Tumor Necrosis Factor-α; PPAR-γ, Peroxisome Proliferators-Activated Receptors-γ; PKA, Protein Kinase A; AMPK, Adenosine 5’-Monophosphate Activated Protein Kinase; Fsp27, Fat Specific Protein 27; Fzd, Frizzled; LRP1, Laminin Receptor Protein 1; ADGRD1, Adhesion G Protein-Coupled Receptor D1; VEGFA, Vascular Endothelial Growth Factor 1; F0-ATPase, F0-ATP Synthase 3 Receptors that bind to PEDF.

## 3 Receptors that bind to PEDF

### 3.1 Adipose triglyceride lipase

The dynamic balance between energy intake and consumption is critical for maintaining the integrity of an organism. When calorie intake exceeds energy expenditure, the remaining energy is converted into fatty acids (FAs) and stored as triglycerides (TGs). However, when energy expenditure (EE) surpasses caloric intake, TG is hydrolyzed and FAs are released (80). Dysregulated lipolysis leads to an overabundance of circulating FAs, which leads to several destructive, lipotoxic pathologic changes, such as insulin resistance, type 2 diabetes, fatty liver, nonalcoholic fatty liver disease, and inflammation (44–47). In 2004, three independent institutes identified ATGL, the main enzyme critical for the initial step of TG degradation, and named it ATGL (81), iPLA2ζ (82), and desnutrin (83). To prevent confusion, we use ATGL in the following discussion. The human gene encoding ATGL is located on chromosome 11p15.5 and includes 10 exons encoding a 504 amino acid protein (NCBI Reference Sequence: NG_023394.1). As a key participant in lipid catabolism, ATGL is highly expressed in brown adipose tissue (BAT) and white adipose tissue (WAT). However, in nonadipose tissues such as skeletal muscle (84), heart (85), liver (86), lung (87), and ocular tissues, and especially in the neural retina and RPE cells (ARPE-19 and hTERT) (39), ATGL mRNA is highly expressed, although at a lower level than in adipose tissues. In addition, researchers found that ATGL was also expressed in human Y-79 cells (11), mouse photoreceptor (661W) cells (88), a rat photoreceptor precursor (R28) cell line (39) and bovine RPE cells (89). ATGL is mainly localized in TG-rich intracellular lipid droplets (LDs), and the catalytic site in human and murine ATGL comprises a dyad consisting of serine 47 and aspartate 166 at the N-terminus of the protein (81, 90). Notably, the following factors may affect ATGL localization: the C-terminal portion includes a hydrophobic LD-binding region, and mutation in the ATGL C-terminus can negatively affect AGTL-LD binding (44). In addition, ATGL binds LC3, an autophagosome marker that promotes ATGL recruitment to LDs (47).

ATGL and hormone-sensitive lipase (HSL) are the major lipases in adipocytes in humans and mice. Several factors are involved in the regulation of ATGL activity. During catecholamine-stimulated lipolysis, for example, PKA phosphorylates α/β hydrolase domain-containing 5 (ABHD5) and promotes ATGL release from the LD surface. The direct interaction of ABHD5 with ATGL promotes the hydrolysis of triacylglycerols (TAG) (91) and broadens region-specificity of ATGL, because as it no longer exclusively engages in sn-2 reactions and engages in more sn-1 and sn-2 reactions (92). Notably, in adipocytes, the combination of an adipocyte- or FA-binding protein with ABHD5 induces ATGL activity, probably by preventing the inhibition of ATGL protease activity (93). In nonadipocytes, ubiquitin regulatory X domain-containing protein 8 (UBXD8) interacts with ATGL and causes the dissociation of CGI-58, resulting in a decrease in ATGL activity (94). ATGL activity is activated by ABHD5 and inhibited by the G0S2 protein in a noncompetitive manner (95). Specifically, G0S2 engages in direct protein-protein interactions and thus impedes substrate accessibility (96). Hypoxia-inducible gene 2 (HIG2), a HIF-1 target, has recently been identified as an inhibitor of ATGL that directly interacts with the AT GL patatin-like phospholipase domain (97, 98).

**Figure 1:**
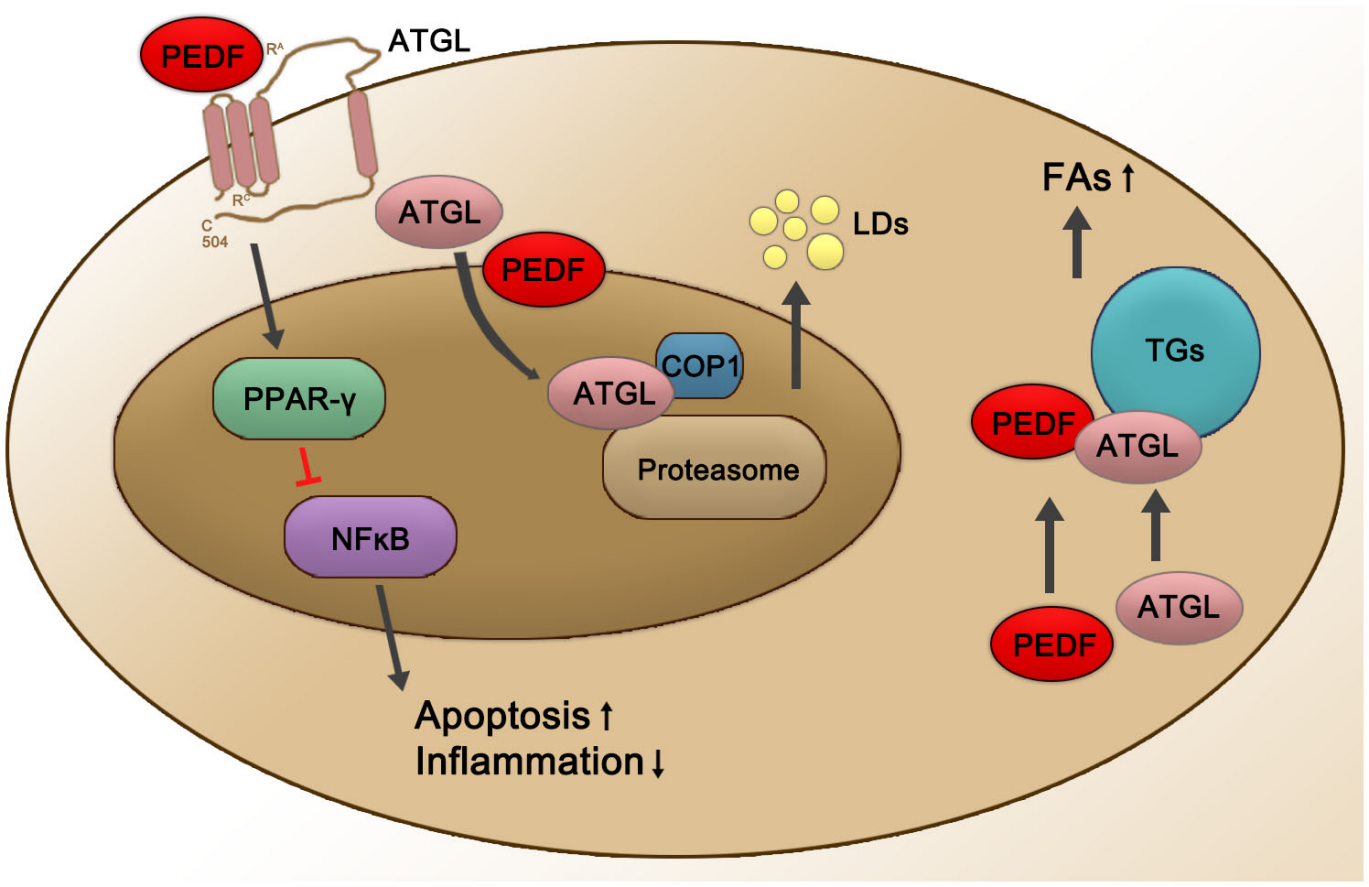
Schematic representation of PEDF-activated ATGL signaling. PEDF binds to the cell surface membrane receptor ATGL, which leads to the upregulation of PPARγ and then suppresses NFĸB at the transcriptional level, subsequently leading to decreased inflammation and an increased apoptosis rates. PEDF also enhances nuclear import of +predominantly cytosolic ATGL protein for its subsequent proteasomal degradation in the nucleus, thereby diminishing ATGL protein stability in a COP1-dependent manner, which enhances the release of LDs. In addition, the PEDF-ATGL interaction promotes the hydrolysis of TGs and the release of FAs. Red lines represent inhibited pathways, and black arrows show activated pathways. PPAR-γ, peroxisome proliferator-activated receptor γ; NFĸB, nuclear factor ĸB; COP1, constitutive photomorphogenic 1; LDs, lipid droplets; TGs, triglycerides; FAs, fatty acids.

PEDF is also involved in regulating ATGL (see **Figure 1**). As a secreted protein, PEDF binds to ATGL on the RPE cell membrane, mediating the first step in its biological effects (33). PEDF regulates lipid metabolic functions through a concomitant elevation in the scavenger receptor CD36. The crucial roles played by PEDF are thought to be mediated through ATGL activation (99). For example, PEDF enhances the nuclear import of the predominantly cytosolic ATGL protein to promote its subsequent proteasomal degradation in the nucleus, thus diminishing ATGL protein stability in a COP1-dependent manner (99). During the development of inflammation-associated hepatic steatosis, endogenous PEDF directly interacted with ATGL and was neutralized by TNF-α, possibly in a PPARα-dependent manner (100). Endothelial damage is critical to vascular leakage and the subsequent shock in sepsis patients. Elevated serum PEDF levels in patients with sepsis induced increased extravasation, which was induced by the abnormal expression of ATGL (101). In addition, both PEDF and the PEDF-derived peptide 44mer promoted cardiac triglyceride degeneration by binding to ATGL (33).

ATGL is regulated by many factors at the transcriptional and post-transcriptional levels. In mice, ATGL controls energy supply and systemically hydrolyzes FAs, thereby influencing mitochondrial β-oxidation and the activation of peroxisome proliferator-activated receptors (PPARs) (102). ATGL decreases PPAR-γ signaling in the brain and suppresses appetite. Additionally, ATGL-deficient humans typically have obesity (103). At the transcriptional level, tumor necrosis factor-α (TNF-α) and insulin decrease ATGL expression, respectively. In this process, PPAR-γ interacts with the ATGL promoter and induces ATGL mRNA expression (104). Under starvation conditions, forkhead box protein O1 (FoxO1) showed increased affinity for the ATGL promoter (105), and this affinity was decreased by an increase in insulin level (106). Regarding the posttranscriptional phosphorylation of ATGL, studies with mice have shown that ATGL phosphorylation at Ser406 either by protein kinase A (PKA) or adenosine 5’-monophosphate-activated protein7 kinase (AMPK) increased ATGL activity (107, 108). In addition, fat-specific protein 27 (Fsp27) reduced ATGL transcript levels by regulating the affinity of early growth response proteins for the ATGL promoter in human adipocytes and thus decreased ATGL-mediated lipolysis (109, 110) (see **Figure 1**).

### 3.2 Lipoprotein receptor-related protein 6

Lipoprotein receptor-related protein 6 (LRP6) was first isolated by Sheryl D. Brown et al., who reported the chromosomal location of LRP6 on human chromosome 12 in 1998 (111). The low-density lipoprotein receptor (LDLR) family comprises cell surface receptors, and several members are involved in numerous signaling pathways and are expressed in various target organs (112). LRP6 is a unique member of the LDL receptor gene family, as indicated by sequence comparisons, and exhibits the closest relationship to the LRP5 gene, a possible therapeutic target for type 1 diabetes (113). Both LRP5 and LRP6 function as coreceptors in the Wnt/β-catenin pathway (111); nevertheless, studies have indicated that LRP5 and LRP6 act distinctly owing to differences in their tissue distribution and affinity for individual Wnt ligands (114). LRP5 and LRP6 are structurally related, exhibiting approximately 71% homology at the nucleotide level, and are broadly expressed in humans, including the hippocampus, renal tubular cells, hepatocytes, intestinal epithelial cells, osteoblasts, and osteoclasts (115). Although LRP5 and LRP6 are functionally and structurally similar, LRP6 is more closely related to glucose and lipid metabolism signaling pathways and plays a more crucial role in developmental processes than LRP5 (116–118).

The human LRP6 (hLRP6) gene is located on chromosome 12p13.2, is 150 kb long and consists of 23 exons. hLRP6 is highly evolutionarily conserved, with negligible differences observed among Drosophila, Xenopus and Mus (119). LRP6 is highly expressed in the heart, brain, placenta, spleen, testes and ovaries but is expressed at low levels in the liver, skeletal muscle, prostate and colon mucosa (111). Mice show LRP6 expression levels in the heart, brain, lungs and kidneys similar to those in humans (111). According to a βgeo reporter activity assay, LRP6 is an endogenous gene and has been detected in all types of developing embryo cells (120).

In mice, LRP6 is essential to the canonical and noncanonical Wnt signaling pathways. When the LRP6 gene carries an insertion mutation and an individual Wnt gene is mutated, *Lrp6^-/-^* homozygous embryos present developmental defects (120). Mice that lack LRP6 genes typically exhibit mid-/hindbrain defects, posterior limb truncations, and abnormal limb patterning, similar to Wnt7a-mutant mice (48). In addition, LRP6 plays an important role in the mouse forebrain (121). In Alzheimer’s disease (AD), a neurodegenerative disorder characterized by deficient cognitive processes and the accumulation of amyloid precursor protein (APP) and amyloid-β, LRP6 mutations are considered to be pathogenic factors. Notably, the conditional deletion of the LRP6 gene in mouse forebrain neuronal cells led to the destruction of synaptic integrity and memory loss in an age-dependent manner, potentially due to increased processing of APP in to amyloid-β (51). In addition, De Ferrari GV et al. showed that in two brain bank data series, a single nucleotide polymorphism (SNP) in exon 18 of LRP6 was associated with Alzheimer’s disease (49).

Recently, a reduction in Wnt-mediated transcription resulting from mutations in LRP6 has been confirmed to lead to several features of metabolic syndrome, including atherosclerosis (50), diabetes (55), hyperlipidemia (50, 53), and hypertension (54), but not obesity. Coronary artery disease (CAD) is usually caused by several risk factors of metabolic syndrome. In 2007, Mani et al. reported on a family with autosomal dominant early CAD, characterized by the features of hyperlipidemia, hypertension, and osteoporosis, which were found to be associated with a short piece of chromosome 12p; specifically, the group identified a mutation in LPR6 that encoded a coreceptor in the Wnt signaling pathway (52). The identified mutation damaged the Wnt signaling pathway *in vitro* because an arginine residue in the epidermal growth factor-like (EGF-like) domain was replaced with a cysteine residue. This finding encouraged the investigators to focus on the relationships between LRP6 and various human diseases, especially atherosclerosis (51).

After the binding of the Wnt ligand, the Fzd/LRP6 receptor and the scaffold protein Dishevelled (Dvl) form a signaling body that is endocytosed. The signaling body induced glycogen synthase kinase 3 (GSK3)-mediated phosphorylation of LRP6 on the PPPSP motif, which initiated the phosphorylation of adjacent S/T clusters through casein kinase 1γ (CK1γ) (122, 123). In addition to GSK3, other kinases phosphorylate the LRP6-PPPSP motif in a Wnt-independent manner. For example, due to the initiation of phosphorylation of cyclin-dependent kinase 14 (CDK14/PFTK1) and its regulators, mitotic cyclin Y and cyclin Y-like 1 (CCNY/CCNYL1), the ability of LRP6 to respond to Wnt ligands is greatest during the G2/M period (123–127). Phosphorylated LRP6 recruits a polyprotein called the “destruction complex”, which includes the scaffold protein Axin and Adenomatous Polyposis Coli(APC) tumor suppressors, the kinases CK1α and GSK3, and the protein E3 ubiquitin ligase β-transduction protein repeat sequence (β-TrCP). In the absence of Wnt ligands, CK1α and GSK3 in the destruction complex phosphorylate β-catenin, inducing β-TrCP-mediated degradation by the proteasome. Wnt/LRP6 signaling inhibits destruction complex activity through at least two mechanisms. First, GSK3 is the product of inhibited LRP6 phosphorylation. Second, the signaling body matures into a multivesicular body, in which the destruction complex is isolated with LRP6. Wnt/LRP6 signaling is enhanced by members of the R-sponge protein (RSPO) family of secreted proteins, which bind to the LGR4/5 receptor and the E3 protein ligase RNF43/ZNRF3 to stabilize the Wnt receptor on the cell surface (54, 128–133).

**Figure 2:**
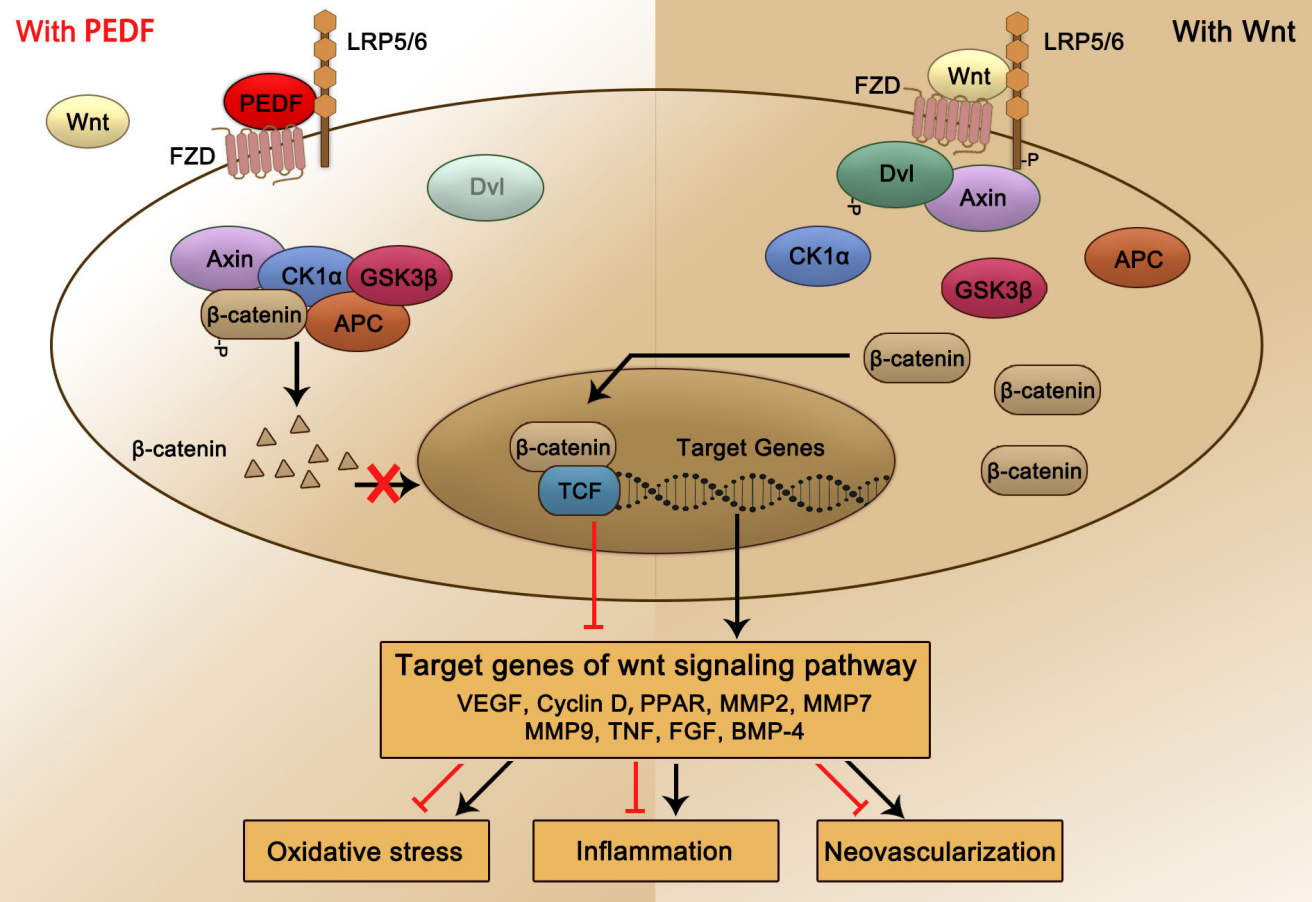
Schematic representation of PEDF-activated LRP6 signaling. PEDF binds to the Fzd-LRP5/6 complex and activates the canonical Wnt/β-catenin pathway. The key protein Dvl is recruited and phosphorylated, subsequently forming a destruction complex that also includes Axin, CKIa, GSK3β, and APC tumor suppressors and inhibiting the phosphorylation and nuclear translocation of β-catenin, which is bound. This process leads to changes in target gene transcription and expression, which reduce the occurrence of relevant pathological consequences, such as oxidative stress, inflammation, and NV. Dvl, dishevelled; CKIa, casein kinase Ia; GSK3β, glycogen synthase kinase 3β.

The canonical Wnt signaling pathway is a conservative intracellular signaling pathway. When the receptor complex on the cell surface, specifically, the Wnt-Fzd-LRP5/6 complex, binds to a Wnt ligand, the canonical Wnt pathway is rapidly activated. Then, Wnt phosphorylation of the cytoplasmic tail of the Fzd-LRP5/6 complex triggers the recruitment and phosphorylation of disheveled protein (also known as Dishevelled, Dvl) in the cytoplasm (134, 135). Subsequently, Axin is recruited to the receptor complex to inhibit its decomposition and the phosphorylation of β-catenin. The stable nuclear translocation of soluble β-catenin in the cytoplasm promotes the activation of the downstream Wnt/β-catenin signaling pathway *via* the transcription and translation of target genes (134, 135). In the absence of Wnt ligands or in the presence of Wnt receptor inhibitors, β-catenin is bound by Axin, Axin and casein kinase Ia (CKIa) and glycogen synthase kinase 3β (GSK3β); moreover, two APC protein kinases combine to form a destruction complex (134). CKIa and GSK3β mediate the phosphorylation of β-catenin in the cytoplasm. Phosphorylated β-catenin is recognized by b-TrCP and induces ubiquitination, which ultimately leads to the degradation of β-catenin (136). Many inhibitors of the Wnt signaling pathway, including Dickkopf WNT Signaling Pathway Inhibitor 1, DKK-1 (137), R-spondins (138), Norrin (139), endostatin (140), PEDF (40), and very low-density lipoprotein receptor, have been identified (141, 142). These negative regulatory molecules of the Wnt signaling pathway ultimately lead to the transcription of target genes, inducing cell proliferation, differentiation, or apoptosis (see **Figure 2**).

### 3.3 Plexin domain-containing 1 and plexin domain-containing 2

Plexin domain-containing 1 (PLXDC1), also known as tumor endothelial marker 7, is a member of the tumor vascular endothelial marker family (143). The genes encoding PLXDC1 are highly expressed during tumor angiogenesis and enriched in many types of tumor endothelial cells (144), including glioblastoma endothelium (57, 63), ovarian cancer (62), gastric cancer (60), renal cell carcinoma (61), colorectal cancer (64), lung cancer (58), breast cancer (56), and osteosarcoma (59). PLXDC1 has been found to be highly expressed in the endothelial cells in the ocular disease DR context and is highly specific to diseased blood vessels (145). PLXDC1 has also been shown to be expressed in rodent retinal ganglion cells (RGCs) (145). Transmission electron microscopy has revealed that PLXDC1 was mainly expressed at tight junctions between endothelial cells, and some PLXDC1 expression was detected on the surface of the blood vessel lumen (146).

PLXDC1 is a novel LRP1-regulated cell-signaling protein. Regulation of PLXDC1 activity by LRP1 was confirmed in CHO cells, and in extracts of RAW264.7 cells and mouse liver, PLXDC1 coimmunoprecipitated with LRP1 (147). The Nidogen/PLXDC1 interaction enhanced cell spreading in PLXDC1-transfected 293T cells, suggesting that Nidogen is a candidate ligand for PLXDC1 (148). Akash Nanda et al. identified cortactin as a protein candidate for binding to the extracellular region of both PLXDC1 and its closest homolog, which were all expressed in the tumor endothelium (146).

Plexin domain-containing 2 (PLXDC2) is a largely uncharacterized transmembrane protein with a homologous nidogen region and a plexin repeat in its extracellular domain. In mice, PLXDC2-betageo expression is prominent in the brain, including in the cortical hem, midbrain-hindbrain boundary, and midbrain floorplate, during midembryonic stages (E9.5-E11.5). On E15.5, PLXDC2 expression was apparent in a large number of discrete nuclei and structures throughout the brain, including the glial wedge and derivatives of the cortical hem. PLXDC2 was also found to be expressed in other tissues, most notably the limbs, lung buds and developing heart, as well as in the spinal cord and dorsal root ganglia (149). In humans, PLXDC2 has been found to be expressed in endothelial cells of the tumor stroma, neural progenitor cells, and pluripotent stem cells and in human hepatocellular carcinoma (HCC) tissues, including HCC cells, tumor vascular endothelial cells, and some infiltrating cells (150).

The PLXDC2 level is elevated during tumor angiogenesis (143). PLXDC2, a mitogen for neural progenitors, has been shown to be a novel regulator of the development of different brain regions involved in the coordinated control of cell proliferation and cell fate along and across the neuraxis (151). PLXDC2 expression increases during cellular senescence and is a marker of adult stem cells (152). In addition, PLXDC2 has been associated with CAD (72), ovarian cancer (73), diabetes mellitus (71), DR (68), testicular germ cell tumors (74), Sotos syndrome (66), ischemic stroke (70), laryngeal squamous cell carcinoma (69), breast cancer (56), and colorectal tumors (65, 67).

**Figure 3:**
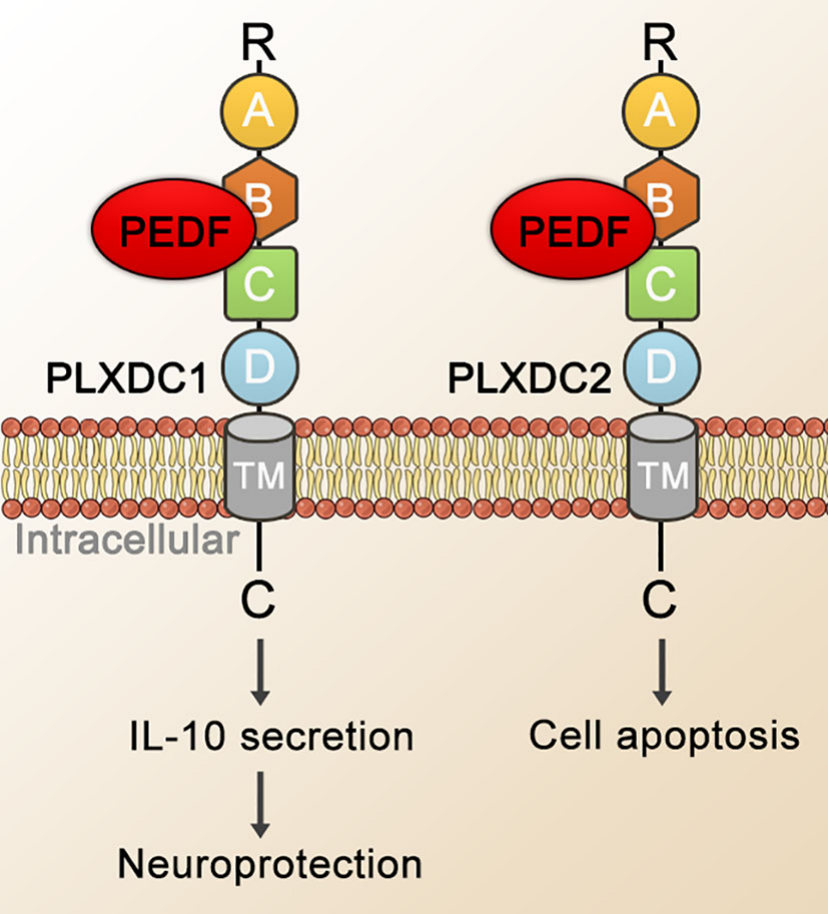
Schematic representation of PEDF-activated PLXDC1 or PLXDC2 signaling. PEDF binds to the cell-surface homologous membrane proteins PLXDC1 and PLXDC2 through their extracellular domains. Domain B plays an important role in binding PEDF. The TM and the C-terminus of these receptors mediate downstream signaling. Ultimately, this signal transduction mechanism stimulates the PLXDC1-dependent secretion of IL-10, which exerts neuroprotective and apoptosis-promoting effects in combination with PLXDC2. TM, transmembrane domain; IL-10, interleukin-10.

In 2014, Guo Cheng et al. identified PEDF binding to PLXDC1 and PLXDC2 on the surface of 293T cells (see **Figure 3**). PEDF interacted with PLXDC1 to promote the secretion of interleukin-10 by macrophages, which exerted neuroprotective effects on rat RGCs, and PEDF combined with PLXDC2 promoted apoptosis in endothelial cells (41). With few reports on the role or mechanism of PEDF and PLXDC in DR or neovascular fundus diseases, PLXDC1 has been shown to inhibit cell proliferation and invasion in tumor cells, promote endothelial cell separation in mice. With a protective effect on RGCs, PLXDC1 is likely to be an important receptor for PEDF, particularly in fundus diseases. In addition to PEDF, other molecules are ligands of PLXDC1 or PLXDC2. Moreover, members of the Wnt family (Wnt3a, Wnt5a and Wnt8b) show a striking overlap with PLXDC2 expression in certain areas (69). Cortactin can bind to the extracellular terminus of PLXDC2 (152). PLXDC2 has also been shown to be an activating ligand for ADGRD1 on cumulus cells (153).

### 3.4 Laminin receptor

The laminin receptor (LR) is a laminin-binding protein with a molecular weight of approximately 67 kDa and is a nonintegrin cell surface receptor of the extracellular matrix (154). LR contributes to laminin binding, ribosome biosynthesis, cytoskeletal organization, and nuclear function, and it controls key cellular processes, including cell adhesion, migration, proliferation and survival, protein synthesis, development, and differentiation (76). Research on LR is mainly focused on its role on the development of tumors, specifically its promotion of tumor cell adhesion to the basement membrane, which is the first step in the tumor cell invasion and metastasis. Therefore, compared with normal cells, LR in tumor cells is overexpressed, and its overexpression is considered to be a molecular marker of cancer metastasis and aggressiveness, including breast cancer, lung cancer, ovarian cancer, prostate cancer, gastric cancer, thyroid cancer, leukemia and lymphoma (75, 76).

**Figure 4:**
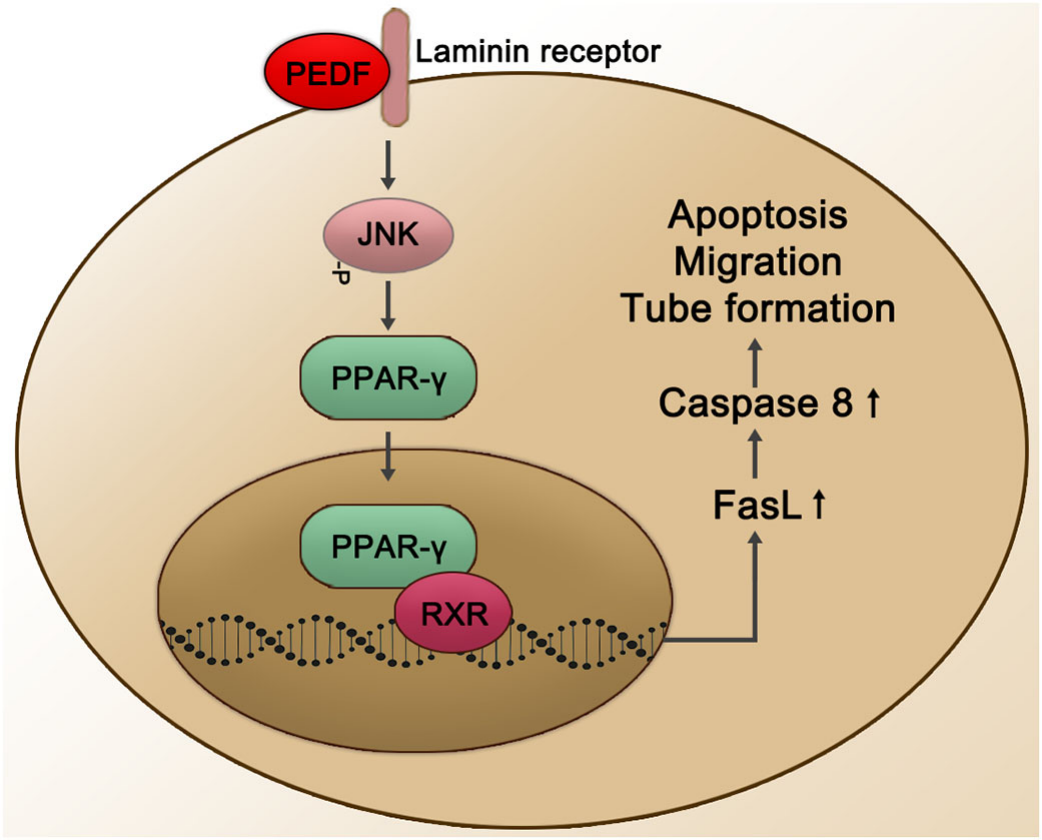
Schematic representation of the PEDF-activated laminin receptor. The PEDF 34mer binds to LR to activate the JNK-PPAR-γ-dependent signal transduction pathway. Subsequently, the combination of PPAR-γ and RXR is activated in the nucleus, promoting the transcription mechanism of FasL gene promoters and affecting biological processes through the upregulation of Caspase 8, such as cell apoptosis, migration, and tube formation. JNK, Jun N-terminal kinase; RXR, retinoid X receptor.

*In vitro* yeast two-hybrid screening experiments confirmed that LR is a cell membrane surface receptor of PEDF. Researchers have also determined that the PEDF isoform that interacts with LR consists of 34 peptides. Surface plasmon resonance analysis showed that PEDF interacts with LR, and PEDF also interacts with endothelial cells. LR-plasma membrane binding induces endothelial cell apoptosis and inhibits endothelial cell migration and the formation of vascular networks *in vitro* (25). The combination of the PEDF 34mer and LR may also mediate endothelial cell apoptosis through the LR-JNK-PPAR-γ-FasL-caspase 8 pathway, thereby exerting an anti-NV effect in the retina. In apoptotic cells, the PEDF 34mer binds to LR to phosphorylate JNK/MAPK, which then activates the transcription factor PPARγ through PPARγ ligand binding and nuclear translocation. In the nucleus, PPAR-γ and RXR combine in the form of heterodimers and recruit specific nuclear coactivators that activate the transcription of target FasL genes in a ligand-dependent manner. Both PPAR-γ and RXR promote the transcription mechanism initiated at FasL gene promoters, ultimately inducing the expression of FasL protein (155) (see **Figure 4**).

### 3.5 VEGFR2

Three VEGFR subtypes are encoded by different genes: VEGFR1 (Flt-1 in mice) and VEGFR2 (Flk-1; KDR) are structurally similar, while VEGFR3 (Flt-4) carries proteolytically cleaved extracellular domains (156–158). VEGF receptors are highly expressed in endothelial cells, hematopoietic cells, macrophages, and smooth muscle cells (159–161). VEGF-A isoforms drive physiological and pathophysiological angiogenesis *via* VEGFR1 and VEGFR2 signaling, while lymphatic angiogenesis is mediated by VEGF-C/D isoforms *via* VEGFR3 (162). VEGFR2 is a large membrane protein consisting of seven extracellular immunoglobulin (Ig)-like domains, a single transmembrane helix, and a split intracellular kinase domain. VEGF-A is an endogenous agonist of VEGFR2 that binds to ligand binding sites in Ig-like domain 2 (D2) and D3 on the basis of the stoichiometry of one VEGF-A dimer to one VEGFR dimer (163, 164).

VEGF-A, VEGFR1, and VEGFR2 play important roles in physiological and pathological angiogenesis, including tumor angiogenesis. Although VEGF-A isoforms exhibit similar VEGFR2-binding properties, activation of VEGFR2 is a complex multistep process. The VEGFR2 signaling pathway is critical for controlling vascular endothelial cell activity, migration, and permeability (165, 166). In cancer cells, transcription factors induced by hypoxia-inducible factors rapidly increase gene transcription rates, resulting in elevated levels of VEGF gene expression while enhancing the stability of VEGF mRNA (78, 79). The direct result of VEGF-VEGFR binding is endothelial cell budding, increased vascular permeability, and increased expression of tissue matrix metalloproteinases, resulting in extracellular matrix digestion. These processes lead to the movement of endothelial and pericyte cells, eventually leading to vasodilation and the formation of pathological vascular networks.

In 2006, Jianxing Ma demonstrated that PEDF inhibited VEGFR2 (167). PEDF inhibits VEGF expression at the transcriptional level by inhibiting hypoxia-induced increases in VEGF promoter activity, HIF-1 nuclear translocation, and mitogen-activated protein kinase phosphorylation. Other investigators have reported that PEDF exerts the anti-VEGF actions by binding to its key angiogenic receptor, VEGF-R2 (43). This binding inhibits receptor activation and ultimately reverses critical downstream VEGF-A signaling by reducing VEGF-R2 tyrosine kinase activity and inhibiting two phosphorylation sites in VEGF-induced HUVECs (Y951 is critical for cell migration and pathological angiogenesis, and Y1175 phosphorylates downstream signaling). Inhibition of VEGF angiogenesis signaling molecules such as PI3K, PLC-γ, FAK and Src may regulate many aspects of endothelial cell biology, including cell proliferation, migration, and survival. Behzad Shahbazi et al. also showed that PEDF binds to the VEGFR2, as well as laminin receptor much stronger than ATP synthase β-subunit, VEGFR1, and LRP6 using molecular docking, molecular dynamics (MD) simulation, and Molecular mechanics/Poisson-Boltzmann surface area (MM/PBSA) approach. Besides, the N-terminal of PEDF owns the most important enactment in the interaction with its receptors (168).

### 3.6 F1-ATP synthase

F-ATP is an ATP synthase in the bacterial plasma membrane, the eukaryotic mitochondrial inner membrane and chloroplast thylakoid membrane. F-ATPase leverages the membrane proton gradient to drive ATP synthesis, passing the passive proton flux through the membrane along its electrochemical gradient, and releasing newly formed ATP from the active site of F-ATP synthase using the energy released by transport reaction (169). F-ATP synthase is composed of two domains, F0-ATP synthase and F1-ATP synthase. In previous studies on angiogenesis, lipoprotein metabolism, innate immunity, hypertension or food intake regulation, F1-ATP synthase was found to be expressed only in cell mitochondria and was used to synthesize ATP that was needed by cells(77). Luigi Notari et al. demonstrated for the first time that PEDF binds F1 ATP synthase b subunit on the endothelial cell surface and inhibits endothelial extracellular ATP synthesis (170). In addition, F1-ATP synthase can inhibit cell growth, proliferation, and migration and directly induce multiple forms of cell death (171).

## 4 Therapeutic roles of PEDF and its receptors in ocular fundus diseases

Millions of people worldwide are affected by ocular fundus diseases such as DR, age-related macular degeneration (AMD), retinal vein occlusion (RVO), retinal artery occlusion (RAO), and fundus tumors. Inflammation, oxidative stress, neurodegeneration, and neovascularization (NV) are involved in the development of these diseases. As an endogenous multifunctional factor, defects or deficiencies that alter PEDF expression have been shown to be closely related to the progression of some of the abovementioned diseases. Related studies have shown that PEDF levels are markedly decreased in the vitreous, retinas and aqueous humors of diabetic patients with proliferative DR compared with those of either nondiabetic individuals or diabetic patients with nonproliferative DR (18, 24, 172). The high-glucose environment in diabetic patients directly downregulates the expression of PEDF in RPE cells, resulting in the loss of its inhibitory effect on angiogenesis. Other studies have shown that the early postnatal retinal vascularization occurs at a faster rate in PEDF-deficient mice, and NV has been observed in the OIR model (26, 38). Injection of exogenous PEDF into the vitreous can significantly inhibit this pathological change (173). These studies have shown that an increase or decrease in PEDF expression is likely important to the development of retinopathy of prematurity (ROP) lesions and abnormal vascular changes. Notably, Holekamp et al. and Park et al., studying neovascular age-related macular degeneration (nAMD) patients and an argon laser-induced choroidal neovascularization (CNV) model in BN rats confirmed that decreased PEDF expression levels were associated with oxidative damage to RPE cells and may lead to CNV formation, which is a key factor in AMD pathological changes (37, 174). Moreover, studies with proliferative vitreoretinopathy (PVR) patients have shown increased levels of PEDF in the vitreous (175), suggesting that PEDF may act through a positive regulatory feedback loop to counteract the angiogenic response and inhibit the activity of fibrotic factors. The dynamic changes in PEDF levels in the abovementioned case studies suggested its potential therapeutic role in related fundus diseases. Clarifying the mechanism and molecular pathway of signal transduction mediated or induced by the membrane surface receptor of PEDF will contribute to the in-depth understanding of the pathological mechanism of fundus lesions and provide new ideas for disease intervention and treatment.

There are extensive reports on the role of PEDF and ATGL in ophthalmology. As mentioned above, ATGL is expressed in a variety of cells in the eye. In the natural retina, ATGL is distributed in the RPE, the inner segments of photoreceptors, and inner nuclear and RGC layers (39). Western blots of ARPE-19 cells and R28 cells membrane fractions yield a single 83-kDa band representing an immunoreactive protein. Specific biotinylation of cell-surface proteins enables ATGL labeling in ARPE-19 cells, and kinetic experiments have shown that fluorescein-labeled human recombinant PEDF binds to cell-surface proteins with a structure similar to that of ATGL. Based on these findings, ATGL is considered to be a plasma membrane protein that interacts with PEDF in these retinal cells (39). ATGL shows phospholipase A2 (PLA2) activity, which is enhanced after binding to PEDF, resulting in the release of active nonesterified fatty acids (NEFAs) and lysophosphatidic acid (LPA), which can be a second messenger or interact with C protein-coupled receptors (107). PEDF increases the PLA activity of ATGL, which plays a potential role in regulating the survival-promoting effect of PEDF on R28 cells (39). Retinal NV is a factor in a variety of eye-blinding diseases, such as DR and ROP. PEDF possibly exerts its antiangiogenic effects by interacting with ATGL in endothelial cells, as indicated by PEDF upregulation of FasL expression by regulating NF-κB in a PNPLA2-dependent manner (102, 103). Ocular neurodegenerative diseases such as glaucoma, hypertensive and ischemic retinopathies are results of the death of RGCs. Müller cells endow neonatal RGCs with trophic properties, and Müller cell-derived PEDF is secreted to support the regeneration of damaged RGCs. Researchers have shown that PEDF secreted by Müller cells promoted RGC survival through the engagement of ATGL (104). R28 cells express neuronal genes and carry functional cell-surface ATGL. PEDF specifically binds to the L4 region in ATGL, which is between Ile193 and Leu249, and thus protects R28 cells from undergoing apoptosis. In other words, ATGL, especially its L4 ectodomain, is essential to the anti-apoptotic and survival effects of PEDF on retinal cells (105).

LRP6 is one of the most widely studied receptors of PEDF, and it plays an important role in the occurrence and progression of DR. Notably, compared with that in nondiabetic controls, the activation of the Fzd-LRP5/6 coreceptor complex in the retina of patients with nonproliferative DR was significantly increased. Studies have shown that in two different recently developed ocular vessel models: OIR mice, which develop ischemia-induced retinal NV, or *Vldlr^−/−^* mice, which develop subretinal NV, the Wnt signaling pathway was activated, and β-catenin was translocated into the nucleus and negatively correlated with the retinal PEDF level (40). Intravitreal injection of PEDF significantly inhibited Wnt signaling pathway activation in this mouse model retina by binding to LRP6 with high affinity and subsequently regulating the expression of downstream target genes, including VEGF, PPAR, MMP2, MMP9, TNF, FGF and BMP-4. As a result, the increase in the oxidative stress response, the increase in the inflammatory response level and the formation of retinal NV in the pathogenesis of DR were attenuated, demonstrating a therapeutic role in DR.

The function and mechanism of PEDF action in fundus diseases mediated through other receptors have not been fully studied. Recent studies, however, have shown that in animal models of retinal NV (an OIR model and a laser-induced CNV model), both the PEDF 34mer and PEDF polypeptide 336 (PEDF 336) exhibit high affinity for LR (176). In a CNV model, PEDF 34mer spot treatment significantly inhibited the formation of CNV through its interaction with LR. In the OIR model, PEDF 336 bound LR with high affinity and inhibited retinal NV (176). In choroidal and retinal endothelial cells, VEGF induced LR expression, which can be inhibited by PEDF 336 (177). In addition, although the roles and mechanisms of PEDF and PLXDC in the eye have rarely been reported, considering that PEDF-activated PLXDC1 protected rat RGCs and that the combination of PEDF and PLXDC2 induced apoptosis of endothelial cells (41), these two membrane receptors may be important to PEDF effects on fundus diseases. They are therefore worthy of further study by researchers. The correlation between VEGFR2 activation and NV has been widely recognized. Zhang et al. proposed that the competitive blockade of VEGF-VEGFR-2 binding by PEDF may be crucial for PEDF effects on VEGF-induced permeability and angiogenesis in retinal capillary endothelial cells (167). Besides, PEDF bound specifically to the extracellular domain of VEGF-R2 and prevented activation of an intrinsic tyrosine kinase, disrupting the flow of VEGF-A downstream signals in primate retinal vascular endothelial cells (43). Furthermore, F1-ATP synthase inhibited cell growth, proliferation, and migration by inhibiting extracellular ATP synthesis and directly induced death in several cell types (19). Therefore, the interaction between PEDF and F1-ATP synthase may reasonably explain for the inhibitory effect of PEDF on NV (42).

## 5 Conclusion

PEDF receptors are widely expressed in ocular tissues and exhibit important physiological and pathological significance. These characteristics implicate the importance of basic research and clinical application of PEDF in the diagnosis, pathogenesis and drug development of DR. DR is a preventable but incurable type of blindness. At present, intravitreal injection of anti-VEGF drugs is an effective treatment method to control retinal NV in patients with DR. Among the many types of anti-VEGF drugs, monoclonal antibody anti-VEGF drugs (such as bevacizumab and ranibizumab) target VEGFR2 and have been designed to antagonize the binding of VEGF and VEGFR2. However, these anti-VEGF treatments still present certain limitations. A number of clinical studies have shown that the effect of anti-VEGF treatment in the clinic was clearly inferior to that obtained in Phase III clinical trials. After intravitreal injection, vision initially improved compared with that at the baseline, but it continued to decrease, not maintaining the initial benefits. Moreover, not all patients responded to anti-VEGF treatment. Exploring new treatments for retinal NV, reducing the number of intravitreal injections by optimizing treatment methods, and administering sustained-release preparations/eye drops have always been the research focus of ophthalmologists and scientific researchers.

Since it was discovered in 1989, PEDF has shown great potential as a drug target for the treatment of DR due to its antiangiogenic, anti-inflammatory, neuroprotective, and neurotrophic effects. With increasing attention on PEDF and the gradual deepening of relevant exploration, an increasing number of researchers have focused on mechanistic research, drug development and clinical transformation of PEDF-specific peptides with biological effects and have clarified the high-affinity structure of PEDF and PEDF receptors. Domains and specific amino acid sequences are helpful to locate PEDF-derived peptides that can truly perform a certain biological function. The therapeutic effect of PEDF-derived peptides is clear and focused, and as a therapeutic drug, its administration method is more adaptable and efficient. For example, drug loaded exosomes or nanomaterials exert therapeutic effects of the PEDF polypeptide to a greater extent than other methods. Therefore, it is important to accelerate the clinical translation and application of PEDF peptides.

Regarding the study of PEDF receptors alone, the roles of LRP6, ATGL and LR in the pathogenesis of DR are relatively clear. For example, the expression levels of LRP6 and ATGL are clearly related to DR pathogenesis, the severity of the disease, and the VEGF expression level. Therefore, the PEDF receptor may be used as a predictive biomarker for the development and progression of DR, which can help in the diagnosis of the disease. In addition, PEDF receptors are expressed not only in eye tissues but also in various tissues of the body, such as heart, bone marrow, kidney and so on. The study of the mechanism of PEDF receptors in nonocular diseases can broaden the horizons of researchers and help their exploration into DR pathogenesis more comprehensively.

There are still certain limitations to the development of treatments that target PEDF receptors. PEDF binds to different receptors and may exert the same effect. For example, by binding to ATGL or LRP6, PEDF exerts anti-inflammatory effects. If ATGL is blocked, LRP6 may continue to compensate and have the inflammatory functions. In addition, most PEDF receptors are not specific receptors and blocking these receptors will affect the physiological functions of other ligands bound to them.

Besides, although PEDF has shown strong therapeutic potential in the ophthalmology field, only one Phase I clinical study has shown that an adenovirus carrying PEDF exerted a therapeutic effect in patients with advanced neovascular age-related macular degeneration. To mediate a biological effect, PEDF must first be ligated to its cell membrane surface receptor. Moreover, PEDF binds to a specific domain of the PEDF receptor or a regulatory site to mediate its biological function. Through the identification of these binding sites, the true value of receptor research in the development of corresponding DR treatments can be demonstrated.

## Author contributions

MX designed this study. MX and XC collected the data and wrote the manuscript. ZY collected the details of tables, figures, generated the tables, figures, figure legends, and wrote the first draft of the manuscript. XL reviewed and revised the manuscript and was also involved with the manuscript development, proofreading and approved the final version of the manuscript. All authors contributed to the article and approved the submitted version.

## Glossary

**Table:**

ABHD5	α/β hydrolase domain-containing 5
AD	Alzheimer’s disease
ADCAD2	Coronary Artery Disease, Autosomal Dominant 2
ADGRD1	Adhesion G-protein coupled receptor D1
AMPK	Adenosine 5’-monophosphate-activated protein kinase
APC	Adenomatous Polyposis Coli
APP	Amyloid precursor protein
ATGL	Adipose triglyceride lipase
ATP5F1	ATP Synthase F1
BAT	Brown adipose tissue
BMP-4	Bone morphogenesis protein 4
CAD	Coronary artery disease
CCNY/CCNYL1	Cyclin Y and cyclin Y-like 1
CD309	Cluster of Differentiation 309
CDK14	Cyclin-dependent kinase 14
CHO	Chinese hamster ovary
Chr	Chromosome
CK1γ	Casein kinase 1γ
CKIa	Casein kinase Ia
CNV	Choroidal neovascularization
COP1	Constitutive photomorphogenic 1
D2	Domains 2
DKK-1	Dickkopf-1
Dvl	Dishevelled
E	Embryonic day
EE	Energy expenditure
EGF-like	Epidermal growth factor-like
F0-ATPase	F0-ATP Synthase
FAs	Fatty acids
FGF	Fibroblast growth factor
Flk-1	Fetal Liver Kinase 1
FoxO1	Forkhead box protein O1
Fsp27	Fat specific protein 27
Fzd	Frizzled
G0S2	G0/G1 Switch 2
GCL	Ganglion cell layer
GSK3	Glycogen synthase kinase 3
HCC	Human hepatocellular carcinoma
HIG2	Hypoxia-induced Gene 2
HIG2	Hypoxia-inducible gene 2
hLRP6	human LRP6
HSCs	Hematopoietic stem cells
HSL	Hormone-sensitive lipase
Ig	Immunoglobulin
IL-10	interleukin-10
iPLA2ζ	Independent Phospholipase A2-ζ
JNK	Jun N-terminal kinase
KDR	Kinase Insert Domain Receptor
LAMR	Laminin Receptor
LC3	Light Chain 3
LDLR	Low-density lipoprotein receptor
LDs	Lipid droplets
LPA	Lysophosphatidic acid
LR	Laminin receptor
LRP1	Laminin Receptor Protein 1
LRP6	Lipoprotein receptor-related protein 6
MD	Molecular dynamics
MM	Molecular mechanics
MMP2	Matrix metallopeptidase 2
nAMD	neovascular age-related macular degeneration
NEFA	Non-esterified fatty acids
NFĸB	Nuclear factor ĸB
NV	neovascularization
OIR	Oxygen-induced retinopathy
PBSA	Poisson-Boltzmann surface area
PEDF	Pigment epithelium-derived factor
PKA	Protein kinase A
PLA2	Phospholipase A2
PLXDC	Plexin domain containing
PNPL2	Patatin-Like Phospholipase Domain Containing 2
PNPLA2	Patatin-like phospholipase domain-containing protein 2
PPARs	Peroxisome proliferators-activated receptors
PPAR-γ	Proliferator-activated receptor-γ
PVR	Proliferative vitreoretinopathy
RAO	Retinal artery occlusion
RCL	Reactive center loop
RGCs	Retinal ganglion cells
RNV	Retinal neovascularization
ROP	Retinopathy of prematurity
RPE	Retinal pigment epithelial
RPSA	Ribosomal Protein SA
RSPO	R-sponge protein
RVO	Retinal vein occlusion
RXR	Retinoid X receptor
SERPINF1	Serpin family F member 1
SERPINS	Serine protease inhibitor superfamily
SNP	Single nucleotide polymorphism
STHAG7	Selective Tooth Agenesis 7
TAG	Triacylglycerols
TEM3	Tumor Endothelial Marker 3
TEM7	Tumor Endothelial Marker 7
TEM7R	Tumor Endothelial Marker 7-Related Protein
TGs	Triglycerides
TNF-α	Tumor necrosis factor-α
UBXD8	Ubiquitin regulatory X domain-containing protein 8
VEGF	Vascular endothelial growth factor
VEGFA	Vascular Endothelial Growth Factor 1
VEGFR2	Vascular endothelial growth factor receptor 2
WAT	White adipose tissue
β-TrCP	β-transducin repeat-containing protein
